# Interferons in Traumatic Brain and Spinal Cord Injury: Current Evidence for Translational Application

**DOI:** 10.3389/fneur.2018.00458

**Published:** 2018-06-19

**Authors:** Francesco Roselli, Akila Chandrasekar, Maria C. Morganti-Kossmann

**Affiliations:** ^1^Department of Neurology, Ulm University, Ulm, Germany; ^2^Department of Anatomy and Cell Biology, Ulm University, Ulm, Germany; ^3^Department of Epidemiology and Preventive Medicine, Monash University, Melbourne, VIC, Australia; ^4^Department of Child Health, Barrow Neurological Institute at Phoenix Children's Hospital, University of Arizona College of Medicine, Phoenix, AZ, United States

**Keywords:** traumatic brain injury, interferon alpha, interferon beta, interferon gamma, interferon alpha receptor, anti interferon alpha antibody

## Abstract

This review article provides a general perspective of the experimental and clinical work surrounding the role of type-I, type-II, and type-III interferons (IFNs) in the pathophysiology of brain and spinal cord injury. Since IFNs are themselves well-known therapeutic targets (as well as pharmacological agents), and anti-IFNs monoclonal antibodies are being tested in clinical trials, it is timely to review the basis for the repurposing of these agents for the treatment of brain and spinal cord traumatic injury. Experimental evidence suggests that IFN-α may play a detrimental role in brain trauma, enhancing the pro-inflammatory response while keeping in check astrocyte proliferation; converging evidence from genetic models and neutralization by monoclonal antibodies suggests that limiting IFN-α actions in acute trauma may be a suitable therapeutic strategy. Effects of IFN-β administration in spinal cord and brain trauma have been reported but remain unclear or limited in effect. Despite the involvement in the inflammatory response, the role of IFN-γ remains controversial: although IFN-γ appears to improve the outcome of traumatic spinal cord injury, genetic models have produced either beneficial or detrimental results. IFNs may display opposing actions on the injured CNS relative to the concentration at which they are released and strictly dependent on whether the IFN or their receptors are targeted either via administration of neutralizing antibodies or through genetic deletion of either the mediator or its receptor. To date, IFN-α appears to most promising target for drug repurposing, and monoclonal antibodies anti IFN-α or its receptor may find appropriate use in the treatment of acute brain or spinal cord injury.

## Interferons: families, signaling and biological properties

Interferons (IFNs) have been historically identified as autocrine or paracrine factors secreted by a large number of eukaryotic cells in response to viral infections, with the ability to effectively restrict the spreading of viruses (1). However, in the last 50 years extensive research has revealed the existence of a large variety of IFN types displaying a panoply of immunomodulatory effects, independent from a strict anti-viral function (2, 3).

There are three distinct types of IFNs. Type-I IFNs include IFN-α (for which 14 genes are known) and IFN-β and the lesser understood IFN-ε, IFN-κ, and IFN-ω. Type-II IFNs include only IFN-γ, which is biologically and genetically distinct from type-I IFNs. A third family (type-III) of IFNs has been more recently described and includes IFN-λ1, IFN-λ2, IFN-λ3 (also known as IL-29, IL-28A, and IL-28B, respectively) and IFN-λ4 (3, 4). The secretion of type-I IFNs is induced in almost every mammalian cell by the exposure to viruses, double-strand RNA or Toll-like receptor activation (5). IFN-γ, in contrast, is released by a number of activated T lymphocytes and subsets of NK cells but also glial cells (6) and is involved not only in antiviral activity but also in the polarization of the immune response and the regulation of macrophage effector functions (7). The family of IFN-λ proteins are expressed only in myeloid and epithelial cells of the skin and mucosae, where they play a role in the maintenance of epithelial barrier integrity and the innate immunity to bacteria, viruses and fungi (8).

Despite being transcribed from independent genes, type-I IFNs share the same receptor, composed of two chains, IFNAR1 and IFNAR2 [Also known as IFN-αR1 and IFN-αR2c; (9)]. However, different isoforms may have slightly distinct binding sites and affinity, which may account for the only-partially overlapping biological effects (4). Upon binding, the dimerization of IFN receptor leads to the phosphorylation and activation of non-receptor tyrosine kinases, Janus Kinase-1 (JAK1) and TYK2, which, in turn, phosphorylate STAT1 and STAT2 transcriptional regulators. Together with the IFN-regulatory factor 9 (IRF9), phosphorylated STAT1 and STAT2 form the IFN-stimulated gene factor 3 (ISGF3) complex, which is directly responsible for the transcriptional response induced by type-I IFNs. Non-canonical signaling from IFNAR1/2 receptor subunits involves the activation of PI-3K/mTOR and MAPK pathways, as well as the phosphorylation of STAT3, STAT4, STAT5A, and STAT5B (2). Conversely, IFN-γ signaling is mediated by a distinct receptor composed of the two subunits IFN-γR1 and IFN-γR2 in a four-chain assembly (10), whose signal transduction cascade involves the activation of JAK1, JAK2 and the phosphorylation of STAT3, STAT5 and the indirect activation of the NF-kB module (11). Type-III IFNs signal through a dedicated receptor formed by the IFNLR1 subunit (also known as IL-28R1) together with the IL-10R2 subunit, which is shared by several cytokine receptors. Type-III IFNs also use JAK1, TYK2, and JAK2 in their signal transduction cascades.

The transcriptional responses elicited by the three types of IFN are remarkably divergent, despite the commonalities in their signaling cascades. Type-I IFNs activate the transcription of genes displaying IFN-stimulated response elements (ISREs) and provide a large-scale regulation of transcription through chromatin remodeling and epigenetic modulation, often in cooperation with other transcription factors [either co-activators or co-repressors; (2)]. Although IFN-γ has been classically related to the transcriptional activation of genes including a Gamma-interferon Activated Sequence (GAS) elements, (12), gene transcription induced by IFN-γ has been shown to recruit multiple transcription factors beyond the canonical STAT [such as C/EBPβ and CREB/AP1; (11)]. The transcriptional responses activated by type-III IFNs are remarkably similar to type-I IFNs and IFN-λ-induced genes and represent a subset of the transcripts activated by type-I IFNs (13, 14).

## Interferons in neurological disorders: pathogenic role and therapeutic applications

IFNs contribute to pathological conditions unfolding in the Central Nervous System (CNS) in often contrasting roles, either as players in the pathogenic process or as therapeutic agents, revealing the far-reaching impact of IFNs in the CNS. A group of genetically determined conditions (mutations in the genes encoding for MDA5, the double-stranded RNA editing enzyme adenosine deaminase ADAR, SAMHD1, the RNase H2 endonuclease complex and the repair exonuclease TREX1), collectively known under the clinical name of Aicardi-Goutieres syndrome (15) is characterized by aberrant production of IFN-α and clinically resembles congenital infections. In fact, astrocyte-restricted overexpression of IFN-α in murine transgenic models results in brain calcifications, gliosis, leukocyte infiltration of meninges and neuronal loss (16). However, infection with the lymphocytic choriomeningitis virus (LCMV) in the same IFN-α overexpression model, results in a significantly lesser degree of damage, inflammation and improved survival. In a different setting involving the comparison of acute and chronic LCMV infection, suppression of type-I IFN signaling by deletion of the *Ifnar* gene (which encodes the IFN receptor shared by all type-I IFNs) ameliorates the clearing of the LCMV and the resolution of the inflammatory response through a mechanism requiring the recruitment of IFN-γ-secreting T lymphocytes (17). Thus, while acute IFN-α may inhibit virus spreading, chronic IFN-α may prevent the transition to an effective immune-cells-mediated clearing of the virus. Thus, IFN-α is pathogenic or protective depending on the underlying condition and the level and timing of expression.

Type-I IFNs have been shown to be involved in the pathogenic cascades of neurodegenerative diseases, whereby IFN-α contributes to the appearance of amyloid-related cognitive deficits in animal models of Alzheimer's Disease (18) and deletion of the *Ifnar* gene has been shown to ameliorate cognitive deficits and attenuate microgliosis. Conversely, deletion of the IFN-β gene in dopaminergic cells results in the appearance of Parkinson's Disease-like pathological features, in particular synuclein aggregates, as a consequence of impaired autophagy (19).

Besides their role in physiology or pathophysiology, type-I IFNs have made a significant impact as therapeutic agents in neurology. The seminal discovery of the therapeutic effect of IFN-β on relapsing-remitting Multiple Sclerosis [MS; (20, 21)] has led to the clinical use of IFN-β as the first disease-modifying drug approved for relapsing-remitting MS. In several large clinical trials (22), IFN-β decreased the rate of clinical progression and reduced inflammatory lesions in the white matter (as detected by MRI). In the last 20 years, a detailed knowledge of the pharmacokinetics, clinical efficacy, and safety of IFN-β have accumulated (23–26), and several variants of IFN-β (with distinct pharmacokinetics) have been developed such as longer half-life pegylated-IFN-β (25, 27). The pharmacodynamics of IFN-β in MS is complex and remains poorly understood. However, type-I IFNs (in particular IFN-β) display a significant anti-inflammatory effect on astrocytes, since treatment of astrocytes with IFN-β results in the induction of an anti-inflammatory transcriptional program orchestrated by the Aryl-hydrocarbon receptor (28). In the EAE MS mouse models, a subset of microglial cells appears to be the major source of IFN-β; exposure of microglia to IFN-β enhances phagocytic activity and loss of IFN-β prevents the clearance of myelin fragments (29). Finally, IFN-β has also been shown to decrease the permeability of the Blood-Brain Barrier (BBB). In fact, deletion of IFN-β in astrocytes facilitated the entry of viruses into the CNS (30). Furthermore, the administration of IFN-β *in vivo* or *in vitro* counteracts the disruption of the BBB caused by inflammatory stimuli (31, 32).

Because of their pivotal role as regulators of neuroinflammation, gliosis and BBB dysfunction, IFNs from all three types may be well positioned to affect the pathogenic cascades in TBI. Although a large number of inflammatory mediators have been reported in the acute neuroimmunological responses to TBI (33) and many have been proposed as possible therapeutic targets (34), only a comparatively small number of studies has addressed the role of IFNs in the pathophysiology of the acute phase of brain or spinal cord traumatic injury (summarized in Table 1).

**Table 1:** Experimental and clinical evidence demonstrating the role of IFNs in neurotrauma.

**Interferon under study**	**TBI model**	**Intervention/genetic deletion**	**Effect**	**Reference**
IFN-α	CCI	IFNAR1-KO	40% decrease in lesion size Reduced IL-1β, IL-6; increased IL-10 Increased M2 microglial polarization	(35)
IFN-α	CCI	IFNAR1 neutralizing antibody MAR1	40–50% reduced lesion size (administered up to 2 dpi) 15–20% improved motor performance	35
IFN-α	EC-Hypp Axonal injury	IFNAR1-KO	Two-fold Increase macrophage infiltration (at 1 dpi) No effect on IL-1β; increased MMP-9	(36)
IFN-α	CCI	miR-155-KO	Reduced (50%) IFN induction Increase (25%) microglial response Increased neuronal loss	(37)
IFN-β	Hemisection SCI	NSC expressing IFN-β	40% decrease in GFAP+ 50% increase in axon preservation modest (1 point) increase in Basso score at 4 weeks	(38)
IFN-β	Weight-drop SCI	IFN-β injection	50% decrease in MPO levels 70% decrease lipid peroxidation 50% decrease in BBB score at 24 h but large spread of the single values.	(39)
IFN-γ	Post-mortem human TBI brain	None	IFN-γ protein IFN-γ mRNA	(40)
IFN-γ	SCI	i.p. administration	Improved motor function Increased accumulation CD1b^+^macroph/microglia Increased MCP-1/CCR3 mRNA Upregulation GDNF & IGF mRNA	(41)
IFN-γ	SCI	IFN-γ-KO	Reduced functional recovery No changes TNF, IL-6, glial responses	(42)
IFN-γ & IFNGR	SCI (contusion)	IFN- γ -KO & IFNGR-KO	No changes	(43)

*The table summarizes the studies reporting the antagonization of various interferon family members in models of neurotrauma, using either pharmacological administration strategies or gene deletion of the IFN or its receptor to determine disease modifying effects. It also includes in situ expression of IFN- γ in post-mortem human brain obtained from TBI victims*.

## IFNs in traumatic brain injury: data from human patients

The expression of IFN-α, IFN-β, and IFN-γ has been investigated in biological samples of human patients, including brain microdialysate, brain tissue and cerebrospinal fluid (CSF). A significant fraction of investigations has focused on validating IFNs as prognostic or diagnostic markers. The main focus has been on IFN-γ because of its well-known role in lymphocyte-driven inflammation. Nevertheless, the recent appreciation of the role of type-I IFNs in inflammation, beyond viral infections, has led to the assessment of type-I IFNs in neurotrauma.

In a small cohort study of 12 patients, 42 cytokines, including IFN-α and IFN-γ, were evaluated in the extracellular fluid of the brain (sampled by microdialysis). Although both IFN-α and IFN-γ were detected in microdialysates, their concentrations varied significantly among the patients and over time, and neither cytokine displayed a reproducible peak in concentration (44). The small size of the patient cohort analyzed, and the intrinsic limitations of cytokine recovery and measurement may have contributed to the inconclusive results.

Quantitative analysis of mRNA levels of IFN-α and IFN-β was performed in post-mortem brain samples obtained from TBI patients (27 patients divided in three cohorts according to their survival after injury: <17 min, <3 and >6 h). Interestingly, the levels of IFN-α transcripts were reduced in samples obtained from patients deceased between 17 min and 3 h after trauma but were comparable to control levels at 6 h or later. Notably, IFN-β mRNA levels were elevated only in samples from patients deceased more than 6 h after TBI and specifically in the injured hemisphere [ruling out an effect of systemic inflammation; (35)].

Changes in IFN-γ, on the other hand, have been investigated in a several clinical cohorts. In a series of patients with severe TBI, the production of eight cytokines were analyzed in the CSF and compared in a group of normoxic individuals (22) to patients suffering from an acute post-traumatic hypoxic episode (20) with the rationale that this severe secondary insult aggravates neuroinflammation, biomarkers of brain damage and long-term outcome (45). When patients were analyzed together (*n* = 42), IFN-γ concentrations were found elevated in CSF at the earliest time point, within 24 h post-TBI and gradually declined to day 5.

Comparison of normoxic and hypoxic TBI patients revealed that both normoxia and hypoxia induced a significant increase in the production of IL-2, IL-4, IL-6, IL-10, GM-CSF, IFN, and TNF, but not IL-8 compared to the control. However, only IFN-γ and GM-CSF were exacerbated by the combination of TBI and post-traumatic hypoxia. In addition, in the hypoxic cohort, the secretion of IFN-γ, and to a lesser extent of TNF, was found to be prolonged up to 4–5 days post TBI compared to the normoxic counterpart. Amplified IFN-γ and inflammation in general, were corroborated by higher levels of the serum injury biomarker S100B and worse outcome scores at 6 months post-TBI using the Glasgow Outcome Scale Extended (GOSE) in hypoxic patients. The secretion of IFN-γ into the CSF is attributed to the upregulation within the injured brain. In fact, in human post-mortem brains, IFN-γ was found significantly overexpressed within a few minutes after TBI, subsequently reaching a >10-fold increase in the brains of individuals dying several hours after TBI. In fact, among the eight cytokines analyzed, IFN-γ reached the third highest elevation after IL-6 and IL-8. Interestingly, the area of the cortex used for cytokine analysis also presented astrogliosis and macrophage activation located in proximity to axonal pathology, implying a direct link between cellular and humoral inflammation (40).

IFN-γ is secreted by glial cells and infiltrating monocytes and is involved in promoting neuroinflammation but also neuroprotective processes such as neurogenesis and brain repair (6). *In vitro* studies have also demonstrated that IFN-γ is a hypoxia-specific mediator induced by T-cells (46). Altogether, these findings suggest that there is an increased secretion of IFN-γ after TBI, its expression and secretion are enhanced after hypoxia and it plays a critical role in secondary brain damage elicited by an acute hypoxic insult following brain trauma. Additionally, IFN-γ plays a critical role in the activation of the kynurenine pathway, which metabolizes the essential amino acid tryptophan leading to the release of the potent neurotoxic factor quinolinic acid, an excitotoxic agonist to the NMDA receptor. In 28 patients with severe TBI some of us reported that critical downstream metabolites of tryptophan were significantly elevated in CSF and that quinolinic acid was higher in the patient cohort with unfavorable outcome and was inversely correlated with the GOSE scores. Furthermore, the overexpression of the upstream enzyme of the kynurenine pathway, indoleamine 2,3-dioxygenase-1 (IDO-1), which is activated by IFN-γ, was detected in post-mortem brains after trauma and associated on tissue pathology (47).

Despite the limitations due to lack of homogeneity between studies in patient selection, cytokine panels and detection methods, converging evidence suggests that all three IFN-α, IFN-β and IFN-γ are induced in human brain after trauma, with different time courses: IFN-α appears to be the first to increase, followed by IFN-γ. However, IFN-α expression appears to be transient, whereas elevation of IFN-γ persists for several days. Because of the complexity of the clinical picture, human studies cannot provide evidence on the role of each IFN in the pathogenic cascade, and consequently on their potential as therapeutic targets. For this purpose, experimental data in murine models need to be evaluated.

## IFNs in traumatic brain injury: experimental evidence

Expression levels of both IFN-α and IFN-β have been verified in controlled cortical impact (CCI) murine model of TBI, whereby the former peaks at 2 h post-injury whereas the latter is not upregulated before 24 h (35). The combined functional role of IFN-α and IFN-β in CCI has been explored in IFNAR1-KO mice since this is a common receptor for both factors resulting in the abolishment of both IFN-α and IFN-β signaling. Notably, in the IFNAR1-KO mice not only is the signaling of IFNs blocked but the transcription of the IFN-α and IFN-β is also reduced, in agreement with the role of IFNAR1 in the positive feedback loop amplifying IFN-α levels and the overall IFN response pathway (48). Overall, upon CCI IFNAR1-KO mice show a significant decrease (40–50%) in lesion size, implying a detrimental impact of type-I IFNs after TBI (35). Mechanistically, loss of type-I signaling results in a significant downregulation of pro-inflammatory IL-1β and IL-6 and in a marked upregulation of the anti-inflammatory mediator IL-10 upon CCI, suggesting a reduced inflammatory response (35). However, 24 h after TBI, both astrocyte reactivity and microglial density are enhanced in IFNAR1-KO mice by 50 and 20%, respectively. In IFNAR1-KO mice, activated microglia expressed high levels of CD206, a marker of trophic M2 macrophages, suggesting that, although increased in number, microglial cells may have assumed a neuroprotective, anti-inflammatory phenotype, in agreement with the upregulation in IL-10. Thus, the IFNAR1-KO data supports the hypothesis that in wild-type mice, type-I IFNs contribute to skew the microglial response toward an inflammatory phenotype, increasing the loss of neurons detected as larger lesion size (35).

Comparable results have been obtained with acute suppression of IFNAR1 via the administration of the anti-IFNAR1 monoclonal antibody, MAR1-5A3. Delivery of MAR1-5A3 before CCI reduced the lesion size by 40%, similar to the effect achieved when the antibody was administered 30 min after trauma. Notably, MAR1-5A3 proved to be efficacious even when administered up to 2 days post-injury (dpi) resulting in a lesion size reduction by 40% and enhanced motor recovery, suggesting an extended therapeutic window for type-I IFNs (35). In agreement with the KO data, treatment with MAR1-5A3 suppressed TBI-induced upregulation of IL-1β, IL-6, and IFN-β, but does not affect the upregulation of IL-10 (35). Although it is not clear whether MAR1-5A3 delivered systemically crosses the BBB, it has been hypothesized that it may act on circulating leukocytes expressing IFNAR1. In fact, chimera mice in which IFNAR1-KO bone marrow was transplanted into a WT recipient causing a specific lack of IFNAR1 only on leukocytes, display a 30–40% decrease in lesion size compared to WT mice, an overall effect similar to what observed in full IFNAR1-KO. In these chimeric mice, the level of microglial activation are actually increased, as observed in IFNAR1-KO, once again suggesting that in the absence of IFNAR1 signaling in leukocytes, microglia activation plays a beneficial role.

Type-I IFN signaling has been shown to regulate the recruitment of leukocytes in a distinct model of acute brain injury, namely the surgical disconnection of the entorhinal cortex (EC) from the hippocampus, leading to the denervation of the Dentate Gyrus and the degeneration of the distal part of the severed EC axons. After injury, the induction of IFN-regulated genes, IRF7 and IRF9 (a molecular signature of type-I IFN pathway activation), was observed from 1 to 7 dpi in microglial cells located in the hippocampus of WT mice but was undetectable in IFNAR1-KO mice (36). The EC-hippocampal disconnection resulted in the accumulation of leukocytes (CD45-bright CD11b^+^ cells) in the hippocampus of WT mice. However, this response was strongly enhanced by 2-fold in IFNAR1-KO mice at 1 dpi. Interestingly, IFNAR1-KO mice displayed reduced levels of the chemokine CXCL10 (and, to a lesser extent, of CCL-2) although, in agreement with the increased leukocyte infiltration, levels of MMP-9 were actually increased (36). Thus, type-I IFNs may not only upregulate local neuroinflammation (toward a detrimental polarization), but also suppress the invasion of immune cells from the periphery.

The effects of downregulating type-I IFNs have been investigated in mice in which the microRNA miR-155 is knocked out, since this is one of the major miRNAs controlling the inflammatory response (49). Upon CCI, the expression of IFN-α and IFN-β is reduced in miR-155-KO mice. In contrast, microglia activation was increased by 25% in miR-155-KO mice together with a reduction in neuronal survival (37). Since miR-155 is strongly expressed in neurons, it cannot be excluded that this effect is unrelated to the suppression of type-I IFNs transcription.

The mechanisms of type-I signaling on neuronal survival have been investigated *in vitro* in an oxygen and glucose deprivation (OGD) model. Upon OGD, IFN-α was strongly upregulated (11-fold) at 2 h whereas IFN-β displayed a milder (2.3 fold) and delayed expression [24 h after OGD; (50)], resembling the time course observed after TBI *in vivo* (35). IFN-α signaling was instrumental in inducing IL-6 and TNF-α secretion in this *in vitro* model, since knocking-down IFNAR1 attenuated both the OGD-induced cytokine upregulation as well as the induction of IFN-α itself. Notably, neuroblastoma cells in which IFNAR1 expression was knocked-down revealed to be more resilient, showing a reduced level of cleaved caspase-3 after OGD (50). Likewise, IFN-α has been reported to have direct pro-oxidative and neurotoxic effects on neurons (51). Thus, based on this *in vitro* model, it can be deduced that IFN-α signaling promotes inflammation in neuroblastoma cells after OGD, leading to an overall detrimental effect on cell survival.

## IFN-α/β in spinal cord injury

The investigations on the role of type-I IFNs in SCI have focused mainly on the potential therapeutic application of IFN-β in acute SCI; however no information on IFN-α is available.

In a seminal work on the effect of IFN-β on SCI, Gok et al. (39) administered IFN-β at a dose of 10^7^IU during trauma (weight-drop after laminectomy), followed by a second dose of 0.5 × 10^7^ IU 4 h later. When evaluated at 24 h post-injury, the spinal cord from rats injected with IFN-β displayed a 50% decrease in myeloperoxidase activity compared to vehicle-treated rats. Furthermore, in contrast to the sharp elevation observed in vehicle treated rats, IFN-β treatment reduced lipid peroxidation to sham levels. IFN-β-treated rats, on average, displayed a trend toward an improved motor recovery, although the large variations did not allow to confirm significant differences. Consistently, IFN-β-treated rats could climb steeper slopes in the Inclined Plane test than vehicle-treated counterparts.

A second study investigating the effects of peripheral-administration of IFN-β after SCI, using a single dose of pegylated-IFN-β, given 30 min after SCI, demonstrated a reduced upregulation of inflammatory cytokines (52). However, among all the cytokine tested, a significant effect was only demonstrated for IL-6 with approximately a 25% decrease at 6 and 24 h post-injury, for IL-18 with a 20% increase at 5 dpi and for IL-10, with a modest increase at 6 h. With IFN-β treatment, no difference was found in the extent of the glial scar formation or spinal cord cavitation, and although a statistically-significant improvement was only observed in open-field test, this effect was limited to the first week after injury, after which no difference existed between treated and untreated rats. Taken together, these two studies suggest that IFN-β may be beneficial in reducing secondary damage after SCI, although more robust data are required to support these findings.

An alternative approach to utilize the beneficial role of IFN-β in SCI and enhance its local delivery has been pioneered by Nishimura et al. (38) by engineering Neural Stem Cells (NSC) to constitutively secrete large amounts of IFN-β. After spinal cord hemisection, NSCs injected intravenously homed within the injury site and displayed a robust expression of IFN-β. The rats injected with IFN-β-secreting cells showed a significant reduction (35%) in astrocyte proliferation and an enhanced preservation of axons (50% more than in NSC secreting beta-galactosidase as control), ultimately resulting in improved motor performance and larger evoked motor potentials 4 weeks after SCI. These effects were markedly reduced when NSCs were depleted by the administration of the cytostatic compound 5-FluoroCytosine (38).

In conclusion, this data suggests that IFN-β may have some beneficial effects in SCI, however the evidence remains limited, possibly due to the restricted CNS penetration of peripherally-administered IFN-β. Thus, more robust experimental data is warranted before IFN-β can be considered as a treatment option in SCI.

## IFN-γ in TBI and SCI

IFN-γ is upregulated in the tissue affected by blunt TBI or in CCI within a time window spanning 2–12 h after trauma (53, 54). Intriguingly, although IFN-γ is an extensively studied cytokine, it has been mainly used as a readout in TBI studies. Substantial literature is available on the genetic or pharmacological manipulations attenuating the upregulation of IFN-γ in TBI (53, 55–58) but very little is known about the role of IFN-γ *per se*. The majority, if not all studies on the subject assumes a pathological role for post-TBI neuroinflammation and therefore by extension IFN-γ must have a detrimental effect. However, this concept has been challenged. In fact, recent evidence suggests that, at least in SCI, the upregulation of IFN-γ may be beneficial (59). In a model of spinal cord contusion, intraperitoneal administration of IFN-γ (1.0*10^4^ UI/day for 14 days), was sufficient to achieve significant levels of this cytokine in the CNS and resulted, unexpectedly, in faster recovery of motor performance from 10 days up to 6 weeks after trauma compared to vehicle-treated mice (41). Interestingly, IFN-γ-treatment did induce a stronger accumulation of CD11b^+^ macrophages/microglia in the spinal cord, but the inflammatory cells were less concentrated in the injury core and more represented in the nearby penumbral and healthy tissue. In agreement with the increased presence of CD11b^+^ cells, the levels of MCP-1 and CCR2 mRNA were upregulated in IFN-γ-treated mice (41). Notably, IFN-γ treatment impacted, unexpectedly on the astroglial response to trauma. Although the activation of astrocytes was increased by IFN-γ treatment, the levels of the chondroitin-sulfate proteoglycans (astrocyte-produced inhibitors of axonal regeneration) were strongly decreased while the levels of GDNF and IGF-I mRNA were upregulated in the injured spinal cord (41).

Interestingly, SCI applied to IFN-γ receptor (IFNGR)-KO mice resulted in worse functional recovery although the local inflammatory response assessed by TNF-α and IL-6 levels as well as astrocyte and microglial responses were not altered (60). In this model, the authors reported that loss of IFNGR resulted in reduced upregulation of adhesion molecules and chemokines in choroid plexus' vascular beds leading to the significant decrease in T lymphocytes in the CSF and in the ependyma as well as in the overall number of CD4^+^ lymphocytes and monocytes in spinal cord at 7 dpi; the authors suggested that at least one of IFN-γ functions in SCI is to facilitate T-lymphocyte and monocyte migration, and, because of the overall detrimental effect of IFNGR-KO on SCI prognosis, have concluded that this IFNGR should have beneficial net effects (60). Similar effects were observed in mice lacking the transcription factor TBX21, which is key to induce IFN-γ transcription (42). Although this data shows that lack of IFNGR does not necessarily improve outcome in SCI, the proposed model may be only one to represent the multiple mechanisms through which IFN-γ affects prognosis in SCI.

In fact, additional evidence on possible beneficial roles of IFN-γ through a distinct, direct T-cell-dependent mechanism has been reported in studies of adoptive lymphocyte transfer in SCI. While transfer of Th1-polarized CD4^+^ lymphocytes enhances neurological recovery after contusive SCI, this effect was significantly attenuated when the transferred lymphocytes were unable to secrete IFN-γ (61). In fact, IFN-γ was found to be a key player in this SCI model by inducing IL-10 production by macrophages and microglia, which, in turn, is the actual effector molecule of IFN-γ beneficial effects. In fact, neutralization of IL-10 abolishes the protective action of IFN-γ-producing lymphocytes.

In contrast to these findings, experimental evidence has also been published detailing a net detrimental role of IFN-γ in SCI. In fact, contusive SCI performed on IFN-γ-KO and IFNGR-KO seems to produce a significantly lower degree of impairment (43). A similar degree of improvement was seen in chimeras in which all bone-marrow-derived cells were IFN-γ-KO. This effect was traced down to a population of T cells expressing γδ TCR whose secreted IFN-γ would act on macrophages to enhance the SCI-associated inflammation and worsen neurological recovery. In fact, chimeras with lack of IFN-γR expression in macrophages as well as adoptive transfer of T-γδ cells unable to secrete IFN-γ, displayed an improved motor recovery after SCI (43). In these conditions, loss of IFN-γ resulted in reduced levels of inflammatory cytokines in the spinal cord and a polarization of macrophages toward the so-called M1, proinflammatory phenotype.

Currently, the divergent results obtained in different studies on the role of IFN-γR/IFN-γ in SCI are not easily reconciled and the issue needs to be revisited taking into account differences in strains and trauma models. It is interesting to note that the level of IFN-γ expression (59, 62) may be an important variable setting the baseline function (mainly inflammatory or anti-inflammatory) in a given mouse strain. Furthermore, the timing of IFN-γ intervention may be critical, since this cytokine may enhance recovery at later stages while still increasing the damage in the acute phase (62). Moreover, the amount of IFN-γ might be affected by additional variables related to trauma, such as hypoxia (46), which should be factored in when assessing the consequence of experimental manipulations of IFN-γ.

Therefore, current data on IFN-γ is not convergent on a specific role of this cytokine. Differences in the amount of the cytokine released and the effects of gene deletion may contribute to these conflicting results. Thus, the translational outlook for targeting IFN-γ in SCI remains unclear.

## Repurposing therapeutic agents to target IFN in TBI and SCI

**Figure 1:**
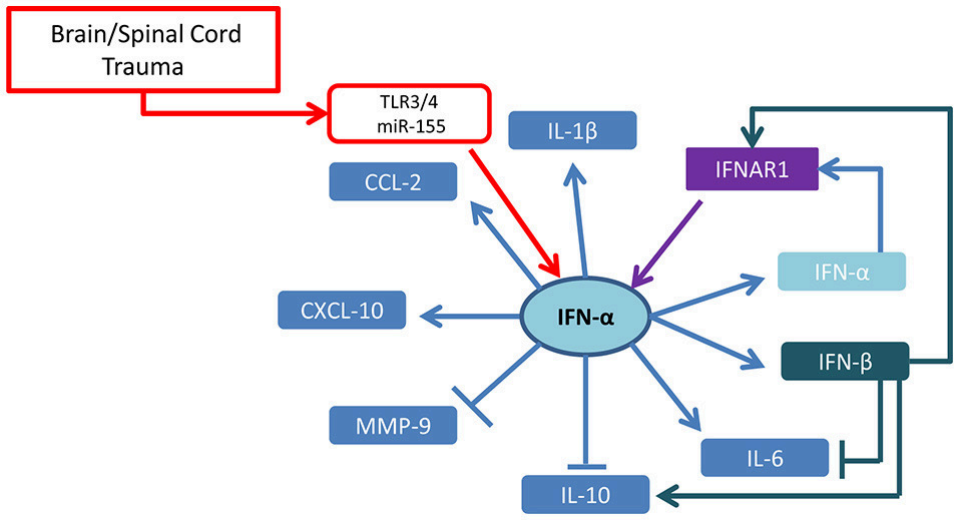
Cytokine network regulated by type-I IFN in brain and spinal cord trauma. Taking into account available evidence based on KO mice and IFN administration in brain and spinal cord injury, the emerging picture shows that IFN-α upregulates its own expression and the expression of IFN-β through the IFNAR receptor and induces CXCL10 and CCL2 chemokines as well as IL-6 and IL-1β. While IFN-α appears to downregulate IL-10, IFN-β administration results in the upregulation of this anti-inflammatory cytokine.

**Figure 2:**
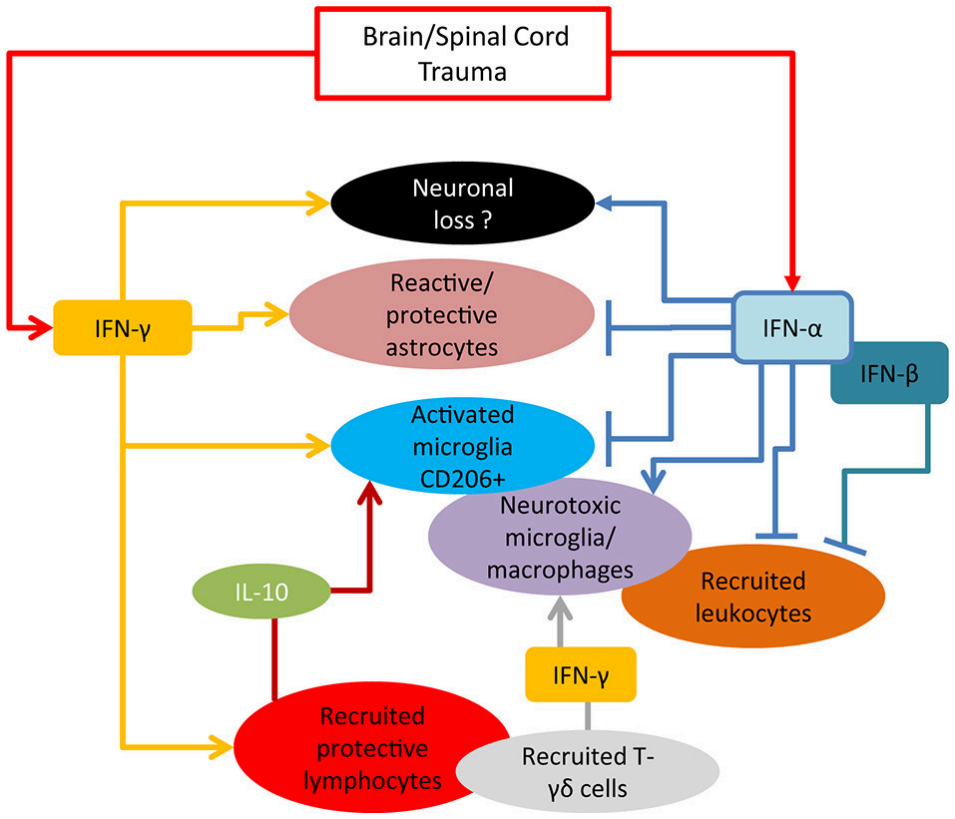
Cellular targets of IFNs in TBI/SCI. Accumulating experimental evidence reported to date mainly derived from KO mouse models, indicates that IFN-α actively limits the acute anti-inflammatory and the reparative responses mediated by microglia and astrocytes, thus favoring a more pro-inflammatory environment. The role of IFN-γ is currently controversial: although able to cause direct neuronal damage and enhance inflammatory neurotoxic cascades (in particular at high concentration), it can also control through the induction of IL-10 the expansion of protective microglia, playing, to this respect, an opposite role to IFN-α.

Type-I and type-II IFNs appear to play distinct and yet not completely identified roles in neurotrauma pathological cascades. Taken together, the current reports suggest that IFN-α seems to be driving the acute inflammatory process through a self-amplification loop and the induction of inflammatory cytokines and chemokines (summarized in Figure 1). On the other hand, IFN-β appears to counteract these effects (at least in SCI model and when administered at pharmacological doses), by upregulating IL-10 and favoring the recruitment of inflammatory cells (cellular targets of IFNs in TBI/SCI are summarized in Figure 2). IFN-γ may be protective or detrimental, possibly depending on the cellular source, the stage of the pathophysiological cascade (acute vs. subacute effects) and the concentration of cytokine released.

**Figure 3:**
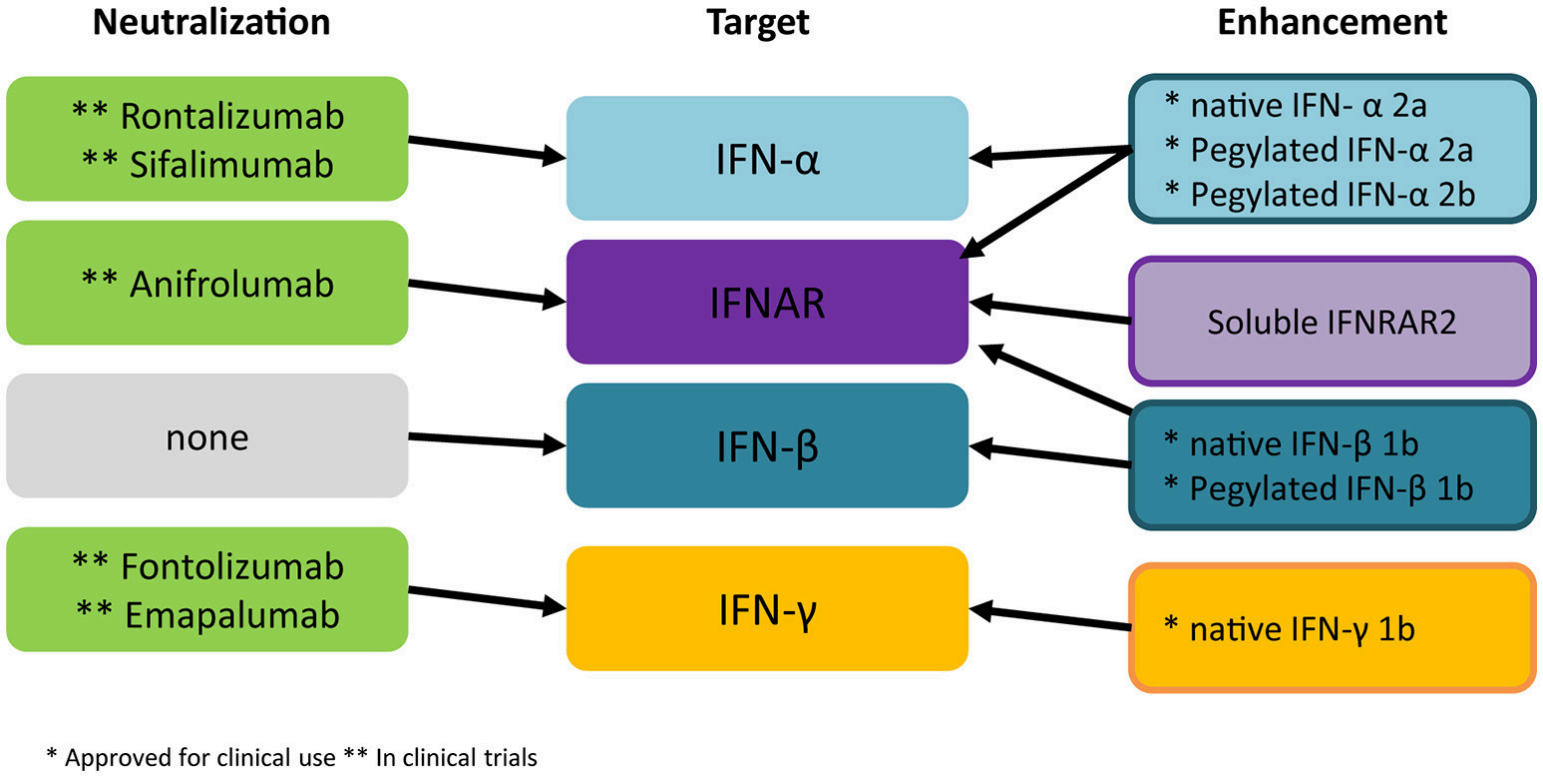
Current strategies for intervention on IFNs. Several monoclonal antibodies directed against IFN-α or the IFNAR1 are currently being evaluated in autoimmune disorders. Experimental evidence suggests that these tools may find application in acute TBI as well. Monoclonal antibodies against IFN-γ have not been successful in clinical applications so far but their role for TBI/SCI treatment should be assessed. All three IFNs have been approved for administration in humans for viral and autoimmune disorders; current evidence points against the use of IFN-α in TBI/SCI, whereas the role of IFN-β and IFN-γ remains to be fully elucidated before administration of recombinant proteins may be considered in human trials. ^*^Approved for clinical use ^**^in clinical trial.

In order to target therapeutically the action of IFNs in TBI two strategies can be taken into consideration: either to administer a specific IFN based on its known beneficial properties or to selectively block a detrimental IFN through the delivery of neutralizing antibodies (summarized in Figure 3).

Indeed, extensive knowledge exists on the administration of IFN-α for the treatment of viral hepatitis and lymphoproliferative diseases as well as the use of IFN-β in multiple sclerosis. The pharmacokinetics of both IFNs have been thoroughly investigated. For instance when administered systemically in pharmacological doses, the penetration of exogenous IFN-α in the CNS through the BBB has been observed (63). In contrast, peripheral delivery of IFN-β appears to be completely excluded from the CNS (64). However, the pharmacokinetics may be significantly different relative to the opening of the BBB, such as in TBI. Recently, the soluble isoform of the IFNAR2 subunit has been found to enhance type-I IFN signaling and to significantly affect pathological conditions. In fact, mice overexpressing IFNAR2 have been reported to be more sensitive to septic shock due to the enhanced IFNAR1 signaling (65), and administration of recombinant IFNAR2 in chronic-progressive Experimental Autoimmune Encephalomyelitis enhances IFN-β signaling, in this case reducing the severity of the disease (66). However, this strategy has not yet been explored for clinical applications since it may enhance detrimental and beneficial effects of IFN signaling with unpredictable effects in TBI.

Despite the high expectations at the time of its discovery, nowadays IFN-γ has limited clinical applications, beside the approval for non-neurological diseases such as Chronic Granulomatous Disease (67), malignant osteopetrosis (68), and as add-on in the treatment of mycobacterial infections. Upon systemic administration, IFN-γ penetrates the brain and spinal cord in significant amounts, although a fraction of IFN-γ in the brain actually binds to the capillary endothelium (69). Thus, being already approved for human use and with known profiles of pharmacokinetics and pharmacodynamics, IFNs would be ideally suited for drug-repurposing efforts in TBI.

Current evidence, based on the deletion of the IFN receptor (35) suggests that enhancing IFN-α signaling by administering IFN-α itself may actually be detrimental, possibly by exacerbating inflammation and gliosis. The effect of administering IFN-β for therapeutic purposes in TBI cannot be assessed because of the deficiency of experimental data on the subject. Nevertheless, the lack of efficacy of IFN-β in SCI (despite some effects on the neuroinflammatory response) makes it an unlikely target for intervention. The data available on the potential of IFN-γ as therapeutic agent is not univocal: although the administration of IFN-γ is beneficial in one setting (41), other authors (43) have shown genetic data suggesting that suppression, rather than enhancement, of IFN-γ signaling may be favorable. Since IFN-γ displays divergent effects depending on the concentration of cytokine available (59), it is possible that a tight control of IFN-γ levels may be necessary to achieve therapeutic success.

Several approaches have been developed to block type-I and type-II IFNs biological actions. In particular, monoclonal antibodies binding to IFN-α such as rontalizumab [a human anti-IFN-α monoclonal antibody that neutralizes all 12 IFN-α subtypes but not IFN-β or IFN-ω; (70)] sifalimumab [fully human, immunoglobulin G_1_ κ monoclonal antibody that neutralizes the majority of IFN-α subtypes; (71)] and IFNAR [anifrolumab, a fully human, IgG1κ monoclonal antibody that binds to IFNAR and prevents signaling by all type I IFN; (72)] have been tested in clinical trials of autoimmune diseases, in particular Systemic Lupus Erythematosus (SLE) and have their safety profiles already investigated (73). Although none of these agents has been tested in TBI clinical settings, the experimental evidence obtained with the MAR1-5A3 (35) suggests that acute neutralization of IFN-α may prove effective. It is unclear how much of the information gained in TBI models (such as CCI) can be transferred to SCI. No experimental data on the neutralization of IFN-α is available for SCI, and the role of IFN-β remains open to question. Therefore, the positive outlook for anti-IFN-α in TBI cannot be extended by default to SCI.

A monoclonal antibody aimed at neutralizing IFN-γ (fontolizumab, a humanized form of a murine anti-human IFN-γ monoclonal antibody) has been developed and tested for the treatment of autoimmune disorders. However, since the efficacy of fontolizumab proved to be disappointing in Crohn's disease (74) and rheumatoid arthritis, its clinical development has not been refined. More recently, a second anti-IFN-γ antibody, emapalumab [a fully human, anti-IFNγ monoclonal antibody; (75)], has entered clinical trials for the treatment of hemophagocytic lymphohistiocytosis (76). To our knowledge, none of these agents is currently scheduled for investigation in TBI or SCI.

The number of biological agents, in particular monoclonal antibodies developed to target specific cytokines has grown exponentially in last few years. In regard to IFNs, there are already available options for either enhancing IFNs by administering recombinant proteins or blocking IFNs using antibodies directed against these cytokines or against their receptors. In light of the current lack of effective therapies for TBI and SCI, the question to be asked for translational applications is no longer “how to target a given cytokine (or mediator)” but rather “which one of the already available therapeutic agents can be repurposed for treatment.” Since drug repurposing offers advantages both to the patients (safer clinical trials, faster entry into clinical applications) and to drug companies (lower development risk, cost and larger return on investment), it is fundamental to provide solid and reproducible rationales to prioritize repurposing efforts. Although both type-I and type-II IFNs appear to be involved in the pathogenic cascade of TBI and/or SCI, their translational outlook appears quite distinct. Evidence available on IFN-β suggests that the net effect of IFN-β administration may be limited. On the other hand, datasets on IFN-γ are inconclusive and both detrimental and beneficial roles have been attributed to this cytokine. Since pharmacological manipulations are available to either increase or decrease IFN-γ levels in humans, it is fundamental to reach a consensus on its role. Current research suggests that blocking IFN-α signaling or neutralizing IFN-α action may offer the best chance for a positive outcome in clinical trials. Since this hypothesis rests on a comparatively limited amount of studies, strengthening the experimental dataset is a priority to advance future translational applications.

## Conclusion: areas of uncertainties

The role of type-I and type-II IFN in acute traumatic injury of brain and spinal cord remains an active area of investigation, in particular because of the opportunity for re-purposing agents whose pharmacology is well understood, either for enhancing or for neutralizing IFNs effects.

In regard to the basic pathophysiology, the main cellular sources of IFNs and the molecular triggers that activate IFNs' responses in TBI/SCI remain poorly understood while the relationship between IFNs and other alarmins, such as IL-33 [shown in other conditions: (77, 78)] has not yet been investigated.

At the translational level, although it appears that IFN-α neutralization is the most promising prospect for successful therapy, little is known about brain penetration of anti-IFN-α monoclonal antibodies already tested in patients and the relative contribution of peripheral vs. central production of IFN-α, supported by the chimeric mouse experiments remains to be fully understood. In addition, because of the long half-life of monoclonal antibodies, it is not known whether prolonged neutralization of IFN-α is necessary or whether its acute and subacute neutralization may lead to different outcomes.

Finally, the diverging roles of IFN-γ must be clarified before any therapeutic strategy could be sketched; in particular, IFN-γ neutralization experiments in TBI have not been fully investigated to date.

Thus, we are still in the early stages of the understanding of IFNs roles in TBI or SCI. As early responders to tissue damage, IFNs are posited to critically influence the acute neuroimmunological response and possibly shape the phenotype and the net effect of the neuroinflammatory cascade in the subacute phase. To this respect, caution must be exerted in extrapolating possible IFNs roles from other diseases or from *in vitro* models, and in assessing critically the reproducibility of reported findings. Therefore, the future of IFNs manipulation for therapeutic purposes must include the spatiotemporal definition of their roles in models that recapitulate as much as possible the anatomical complexity and the physiological peculiarity of the human condition.

## Author contributions

FR and MM-K designed the scope and the structure of the review. FR, AC, and MM-K searched the relevant literature and summarized concepts and results. FR, AC, and MM-K prepared the text and the artwork.

### Conflict of interest statement

The authors declare that the research was conducted in the absence of any commercial or financial relationships that could be construed as a potential conflict of interest.
